# Experimental Florivory Influences Reproductive Success in the Field Bindweed (*Convolvulus arvensis*)

**DOI:** 10.3390/plants15020225

**Published:** 2026-01-11

**Authors:** Pavol Prokop, Adrián Purkart, Juraj Litavský

**Affiliations:** 1 Department of Environmental Ecology and Landscape Management, Faculty of Natural Sciences, Comenius University, 842 15 Bratislava, Slovakia; 2 Institute of Zoology, Slovak Academy of Sciences, 845 06 Bratislava, Slovakia; 3 Department of Zoology, Faculty of Natural Sciences, Comenius University, 842 15 Bratislava, Slovakia

**Keywords:** pollinator preference, pollinator behavior, flower damage

## Abstract

Florivory is the consumption or damage of flowers by herbivorous animals. It can directly affect plant fitness by damaging reproductive organs or indirectly by negatively influencing flower attractiveness to pollinators. We investigated florivory in field bindweed *Convolvulus arvensis* L. (Convolvulaceae) by combining data from natural surveys, experimental damage, and laboratory experiments on flower preferences of florivores. Surveys showed that flowers suffer damage from predators, including *Leptophyes albovittata* Kollar (Orthoptera: Tettigoniidae), which causes partial corolla damage, and from unknown predators that cause holes in the corolla. Experimentally damaged flowers had significantly lower reproductive success (number of seeds and proportion of total reproductive failure) than intact flowers. However, laboratory experiments with naïve bumblebees *Bombus terrestris* L. (Hymenoptera: Apidae) failed to detect a preference for undamaged flowers. This may be because *B. terrestris* is not a frequent pollinator of *C. arvensis* at our field sites, and naïve foragers, lacking prior experience, had not learned to associate corolla damage with reduced floral rewards. Our research shows that florivory negatively impacts *C. arvensis* reproductive success by altering pollinator behavior through reduced flower attractiveness.

## 1. Introduction

Herbivory is a powerful biotic pressure that significantly affects plant fitness across terrestrial ecosystems [1,2]. Herbivores consume plant tissues and reduce seed production [3]. In response, plants shift resources toward defensive systems, lowering their investment in pollinator attraction and reproduction [4]. Herbivore damage is particularly detrimental to plants during the early developmental stages because it compromises future reproduction [5,6]. The experimental removal of herbivores has been shown to increase plant height, flowering, and seed production in some populations [7], providing further evidence for the fitness costs associated with herbivory.

Florivory, or the predation of floral organs [8], is a widespread phenomenon in angiosperms that imposes significant fitness costs, particularly in tropical species, by directly damaging reproductive structures and reducing fruit and seed output [9,10]. However, the effects of florivory on plant fitness are not uniformly negative. Certain studies have shown that plants may exhibit resilience, meaning that florivory itself does not significantly reduce pollination efficiency or plant reproductive success [11,12]. Florivory typically reduces pollinator visits by damaging flower attractiveness [13,14,15]. However, studies on some species have found that pollinators visit damaged and intact flowers equally, with no negative impact on plant fitness [16]. The discrepancy among these findings can be explained by the specific floral structures damaged and the ecological context of the plant–pollinator interaction. Damage to visually conspicuous and attractive structures is more likely to deter pollinators than damage to less critical structures [17]. Furthermore, in species with generalized pollination systems, or high-resource environments, pollinators may not discriminate against damaged flowers, leading to neutral fitness effects. This indicates that the impact of florivory is species-specific and is mediated by complex interactions among floral traits, pollinator responses, and ecological conditions. Ultimately, florivore–plant interactions can drive evolutionary changes by exerting selective pressure on floral traits and reproductive strategies [9].

In this study, we experimentally investigated the impact of florivory on the reproductive success of common field bindweed *Convolvulus arvensis* L. (Convolvulaceae). *C. arvensis* is native to Europe and Asia and grows particularly near agricultural fields and in open, disturbed areas. Hermaphroditic flowers emerge on long twining stems and are pollinator-dependent [18,19,20,21]. The flowers of *C. arvensis* are predominantly visited by beneficial insects such as Hymenoptera, Lepidoptera, and Diptera [18,20,21]. This species is considered one of the most damaging agricultural weeds worldwide [22]. Its vigorous, twining growth allows it to form dense mats that smother crops, leading to substantial yield losses; for example, estimated economic losses in the United States exceeded $377 million in a single year [23]. Furthermore, its extensive deep root system confers drought resistance, but makes eradication difficult. This combination of traits, providing floral resources for pollinators while acting as a severe competitor to crops, makes *C. arvensis* a species of significant ecological and economic interest. Anecdotal records suggest that flowers are the subject of florivory [21]; however, to date, no systematic study has investigated whether florivory impairs reproductive success in this species. To address this gap, we first recorded natural florivory patterns in the field. We then conducted floral damage experiments to test whether corolla damage reduced reproductive success. Finally, we complemented these field data with laboratory experiments using bumblebees *Bombus terrestris* L. (Hymenoptera: Apidae) as model pollinators to examine their behavioral responses to damaged and intact *C. arvensis* flowers.

## 2. Results

### 2.1. Field Surveys

**Figure 1:**
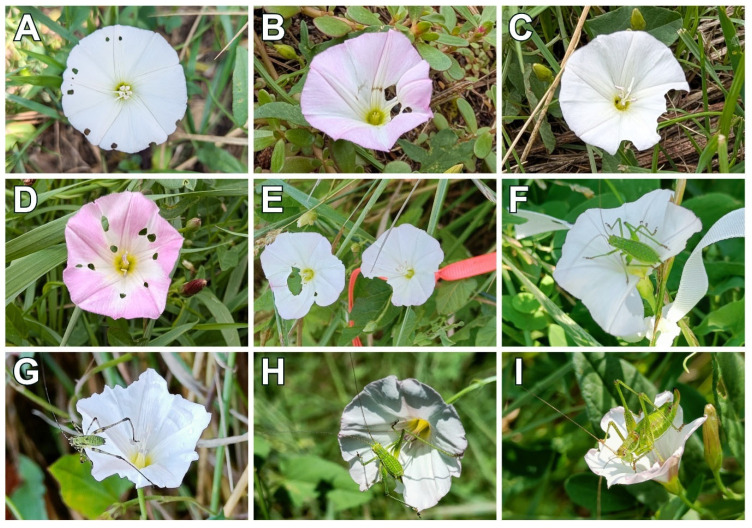
Various types of florivory in *C. arvensis* without (**A**–**E**) and with the predator (**F**–**I**).

We identified very variable patterns of florivory of *C. arvensis* ranging from openings of various sizes (Figure 1A,B,D), to partial corolla destruction (Figure 1C), or both types of predator activity (Figure 1E). *Leptophyes albovittata* Kollar (Orthoptera: Tettigoniidae) was directly identified as a predator of flowers (Figure 1F–I), and this species was responsible for partial corolla destruction. Causes of openings on corollas were not identified.

### 2.2. Field Experiment

#### 2.2.1. Results of GLMM on *C. arvensis* Reproductive Success Measured by Number of Seeds

**Figure 2:**
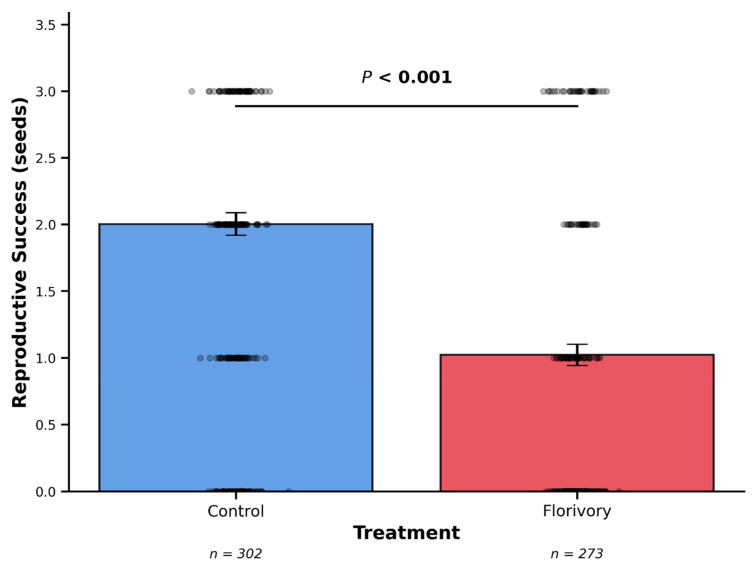
Effect of florivory treatment on reproductive success in *Convolvulus arvensis*. Bars represent mean values, error bars represent standard error of the mean (SE). Individual data points are overlaid (black dots). Statistical significance determined using negative binomial GLMM accounting for clustering by locality.

Treated flowers produced significantly fewer seeds that control flowers (GLMM, estimate = −0.65, χ^2^ = 63.2, df = 1, *p* < 0.001, Figure 2). Florivory treatment reduces reproductive success by ~48%.

The random effects analysis revealed that Locality explained 43.2% of the variance in reproductive success (ICC = 0.432), indicating substantial clustering of outcomes within localities. This suggests that nearly half of the variation in reproductive success can be attributed to differences between localities. In contrast, date explained virtually no variance (ICC ≈ 0.000, variance = 7.46 × 10^−10^), indicating that temporal variation across the four sampling dates was negligible. The residual variance (0.758) represents individual-level variation not explained by locality or date.

#### 2.2.2. Results of GLMM on Total Reproductive Failure of *C. arvensis*

**Figure 3:**
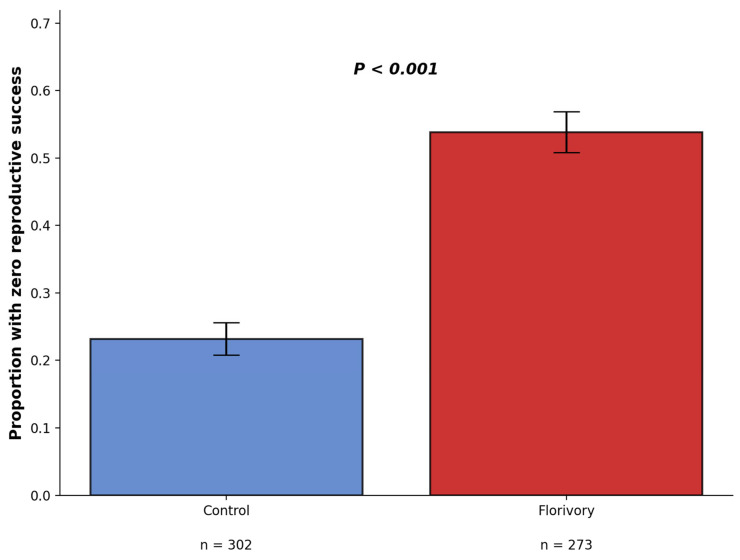
Effect pf experimental florivory on total reproductive failure.

GLMM with binomial dependent variable (capsules obtained 0 seeds or non-zero seeds) showed that the treatment effect was highly significant with (estimate = 1.44, χ^2^ = 58.2, df = 1, *p* < 0.001, Figure 3). Experimental florivory more than doubled the rate of complete reproductive failure—from 23.2% (70 of 302 flowers had empty capsule) in controls to 53.8% in experimentally treated plants (147 out of 273 flowers had empty capsule). These calculations confirm that florivory dramatically increased the probability of complete reproductive failure.

The random effects analysis revealed that Locality explained 34.6% of the variance in the probability of zero reproductive success (ICC = 0.346), indicating substantial clustering of outcomes within localities. This suggests that approximately one-third of the variation in complete reproductive failure can be attributed to differences between localities. In contrast, Date explained virtually no variance (ICC ≈ 0.000, variance = 0.000), indicating that temporal variation across the sampling dates was negligible. The residual variance (3.29, based on the logistic distribution variance of π2/3π2/3) represents individual-level variation not explained by locality or date.

#### 2.2.3. Results of GLMM on Disappearance Rate of *C. arvensis*

The florivory treatment (30/303) showed a numerical trend toward lower disappearance compared with control (49/351) (9.9% vs. 13.96%), but this difference was not statistically significant (GLMM, estimate = −0.28, χ^2^ = 1.22, df = 1, *p* = 0.27).

Analysis of random effects revealed that 25% of the variation in disappearance risk was explained by differences between localities (ICC = 0.25). 0% of the variation was explained by sampling date as the variance component is essentially zero (0.00). Residual Variation was high (74.9%), meaning that the majority of variation (3.29 variance units) was individual-level variation not explained by locality, date, or treatment. This represents flower-to-flower differences within the same locality and date.

### 2.3. Laboratory Experiment

We tested whether bumblebees showed a preference between control and experimentally damaged flowers using a binomial test. Out of 40 choice trials, bumblebees selected control flowers in 22 cases (55.0%) and experimentally damaged flowers in 18 cases (45.0%). Binomial test revealed no significant preference for control flowers (*p* = 0.636, 95% CI: 0.385–0.707).

## 3. Discussion

We used an integrated field and laboratory approach to test the impact of florivory on *C. arvensis*, measuring plant reproductive success in the field and pollinator behavior toward damaged flowers in the lab. Florivory had a significant negative effect on plant reproductive success. However, in laboratory assays using naïve pollinators, visitation behavior remained unchanged irrespective of floral damage. The measured predictors could not reliably explain the disappearance of capsules; therefore, we do not comment further on this issue.

We showed that *C. arvensis* flowers are subject to florivory in the field and identified *L. albovittata* as a predator (herbivore) responsible for the damage. We believe that there are more predators of *C. arvensis* flowers because patterns of florivory caused by *L. albovittata* (Figure 1F–I) differ from the holes shown in Figure 1A,B,D. We speculate that some beetles damage the floral buds before they open. A single puncture to the folded corolla then creates multiple, symmetrically mirrored holes once the flower expands. The identity of the predators (probably *Podonta nigrita* Fabricius, 1794 [Coleoptera: Tenebrionidae] or *Oedemera femorata* Scopoli, 1763 [Coleoptera: Oedemeridae]) [21] requires further investigation of flower buds.

The significantly lower reproductive success of experimentally damaged flowers is most likely explained by reduced pollinator visitation. Since the damage was to the corolla and not the reproductive organs directly, and because *C. arvensis* is pollinator-dependent and fails to set seed without pollinators [21], the most plausible mechanism is that floral damage deterred pollinators. This interpretation is reinforced by the well-documented self-incompatibility of the species [18,19,20,21]. The obligate reliance on cross-pollination makes *C. arvensis* an ideal system for studying florivory, as any reduction in seed set following damage can be attributed directly to pollinator behavior rather than to a compromised capacity for self-pollination in the plant.

The effects of florivory on pollinator behavior and plant fitness are context-dependent. For instance, in *Gelsemium sempervirens* J. St.-Hil., 1805 (Gentianaceae) florivory reduced visits to certain floral morphs while increasing the handling time of others [24]. Similarly, in *Sagittaria lancifolia* L. (Alismataceae) damage by the weevil *Tanysphyrus lemnae* Fabricius, 1792 (Coleoptera: Curculionidae) made female flowers less attractive, directly reducing fruit number, weight, and seeds per fruit [10]. In a complementary experiment on *Nemophila menziesii*, Hook. & Arn. (Hydrophyllaceae) McCall [25] demonstrated that both natural and artificial petal damage reduced floral symmetry and deterred pollinator activity, further supporting the idea that corolla damage alters pollinator behavior. Since florivore damage primarily affected the corolla and not the reproductive organs, the observed reduction in reproductive success in *C. arvensis* is likely a consequence of altered pollinator visitation.

Unexpectedly, our controlled laboratory experiments with bumblebees provided no evidence for the pollinator-mediated mechanism that is often responsible for reduced reproductive success after florivory. We propose several hypotheses to explain this null effect. Firstly, bumblebees are not frequent pollinators of *C. arvensis* (P. Prokop, pers. obs.), but flowers are visited mainly by small halictid bees and syrphid flies [18,19,20,21,26]. Therefore, bumblebees were not an optimal pollinator of *C. arvensis*. Secondly, the bumblebees used in our study were naïve, inexperienced individuals. Experiments with downward and experimentally altered upward flowers of snowdrop *Galanthus nivalis* L. (Amaryllidaceae) showed that naïve bumblebees prefer unnaturally altered upward flowers [27], suggesting that learning significantly affects flower preference in pollinators [28]. Therefore, the behavior of experienced pollinators belonging to different species could be different from that of captive bumblebees. It has been suggested that, at least in certain cases, insects do not discriminate between damaged and intact flowers [16]. Damaged flowers typically offer reduced nectar or pollen rewards, making them less attractive to pollinators [29]. Tattered petals can then serve as explicit cues that insects use for flower avoidance [17]. While some *C. arvensis* morphs display pink radial stripes that could potentially function as nectar guides, the flowers in our study population were predominantly white and lacked contrasting patterns. Therefore, the disruption of visual nectar guides is an unlikely explanation for the reduced pollinator visitation observed. Finally, insects generally respond negatively to asymmetric flowers [29,30], thus avoidance of damaged, asymmetrical flowers can explain our observations [17,31].

## 4. Limitations

A primary limitation of this study is that florivory rates were not assessed in unmanipulated flowers. Published florivory rates are highly variable, ranging, for example, from 9% of flowers [32] to 75% [17]. Our results indicated that predation rates were significantly influenced by locality (explaining up to 43% of the variance), which aligns with prior observations. For instance, Prokop [21] reported no damaged *C. arvensis* flowers in an urban setting, with florivory confined to rural habitats. Additionally, observing the behavior of common pollinators in the field would help collect the data. In summary, additional research on florivory rates is required.

## 5. Conclusions

To conclude, *C. arvensis* flowers are subject to florivory, which alters their appearance in various ways (e.g., holes or partially damaged corollas). Experimentally induced florivory showed that flower predation influences pollinator visitation rates, likely due to negative responses to asymmetrical flowers or to previous experiences with damaged flowers. Future research should prioritize direct observations of pollinator behavior toward naturally damaged flowers in the field.

## 6. Materials and Methods

### 6.1. Field Surveys

This preliminary survey aimed to descriptively analyze the types of physical damage caused by predators on *C. arvensis* flowers. Prior to implementing the experimental design, we conducted qualitative field reconnaissance between 30 July and 20 August 2025. We visited multiple geographically diverse field sites in Western Slovakia where *C. arvensis* occurs. At each site, upon locating a patch of flowering *C. arvensis*, we opportunistically examined plants for the presence of florivore damage, specifically noting any visible injury to the corolla (e.g., notches, holes, or partial removal of the corolla). Examples of damaged flowers, both in the presence of predators and apparently predated, were photographed (see the Results section).

### 6.2. Field Experiment

The experiment was conducted between 30 July and 14 August 2025. We selected four patches with a common occurrence of flowering *C. arvensis* in Slovakia and one patch in Serbia: two patches located 100 m apart were near a corn field (48°39′ N 17°57′ E); two were located along cycle paths (48°13′ N, 17°10′ E and 48°6′8.13″ N, 17°6′27.05″ E); the final patch was located in a garden (45°23′3.30″ N, 20°18′5.36″ E).

**Figure 4:**
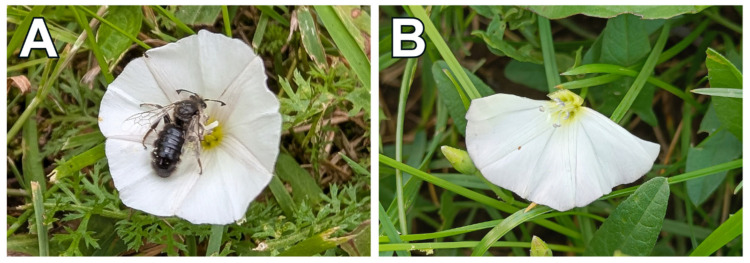
An example of a control (**A**) and experimentally treated flower with half of the corolla removed (**B**). *Systropha curvirostris* pollinates the control flower.

*C. arvensis* flowers (N = 575) were located early in the morning between 6:30 and 7:30 and were marked with a ribbon before opening. The minimum distance between the monitored flowers within the study plots was 20 cm. Approximately half of the flowers were treated by removing half of the corolla using scissors (Figure 4). Flowers were checked for pollination success 14 days later, when capsules had developed or aborted, and seeds were counted [21]. We recorded the occurrence of capsules (presence or absence), the number of seeds in each capsule, and capsule disappearance for each plant. The latter variable likely includes a mix of samples predated by animals and damaged by humans and may not have significant ecological significance. Despite this, we performed statistical analyses on the disappearance rate, given that we could not be certain about the actual causes of the disappearance of some flowers or capsules.

### 6.3. Laboratory Experiment

We used bumblebees (*Bombus terrestris*, L.) as model pollinators to examine the preferences of bees for treated and control *C. arvensis* flowers. A captive colony of naïve bees was obtained from Koppert© (Nové Zámky, Slovakia) and kept at 22–24 °C in a room lit with natural and neon light (370 lx). The bees were connected to a 90 × 50 × 40 cm insectarium using a plastic mesh tube and daily fed exclusively with honey solution (water 60% and honey 40%). The trials started with insertion, placing two freshly collected *C. arvensis* flowers in glass test tubes on the front of the terrarium, 5 cm away from the back wall and 10 cm apart from each other. On a given day, the placement of the flowers was randomly determined (i.e., left or right). When the bee was feeding inside the flower for 3 s, it was considered as successful pollination [33]. Each flower and each bee were used only once. The trials took place 1 week after the colony arrived, in September 2025. All trials (N = 40) took place between 09:00 a.m. and 15:00 p.m.

### 6.4. Statistical Analyses

For the field data, we used Generalized Linear Mixed Model (GLMM) with number of seeds as dependent variable (negative binomial distribution), the effect of treatment (categorical predictor) and date and locality as random effects. Disappeared flowers were not included in analyses.

The disappearance rate (dependent binomial variable) was examined with binomial GLMM with treatment as a categorical predictor and the effect of date and locality as random effects.

For the laboratory data, A two-tailed binomial test was conducted to evaluate whether pollinator preference for control or treated flowers differed significantly from random choice (null hypothesis: *p* = 0.5).

Post hoc power analysis was conducted to evaluate the adequacy of our sample size for detecting the observed treatment effect on reproductive success measured by number of seeds. The analysis was based on a negative binomial GLMM accounting for clustering within localities. The study included 575 observations (302 control, 273 treated) across 5 localities and 4 sampling dates. The observed treatment effect showed a rate ratio of 0.52 (95% CI: 0.3645–0.61). This corresponded to a Cohen’s d of 0.70, representing a medium-to-large effect size. Power analysis revealed that our study achieved 94.6% statistical power (α = 0.05, two-tailed) to detect the observed effect. This high power indicates that our sample size was adequate to reliably detect the treatment effect and minimizes the risk of Type II error. The observed z-statistic of −7.95 (*p* < 0.001) confirms the robustness of the detected treatment effect. The power calculation accounted for the clustered study design and the overdispersion characteristic of count data, using the standard error of the treatment coefficient (SE = 0.08) from the negative binomial GLMM. Given the achieved power exceeding 80%, our study provides strong evidence for the negative impact of florivory on plant reproductive success.

## Data Availability

Data are available as Supplementary Material.

## Supplementary Materials

The following supporting information can be downloaded at: https://www.mdpi.com/article/10.3390/plants15020225/s1.

## References

[1] Jia S., Wang X., Yuan Z., Lin F., Ye J., Hao Z., Luskin M.S. (2018). Global signal of top-down control of terrestrial plant communities by herbivores. Proc. Natl. Acad. Sci. USA.

[2] Pringle R.M., Abraham J.O., Anderson T.M., Coverdale T.C., Davies A.B., Dutton C.L., Veldhuis M.P. (2023). Impacts of large herbivores on terrestrial ecosystems. Curr. Biol..

[3] Mertens D., Douma J.C., Kamps B.B., Zhu Y., Zwartsenberg S.A., Poelman E.H. (2024). Quantifying direct and indirect effects of early-season herbivory on reproduction across four brassicaceous plant species. Funct. Ecol..

[4] Jacobsen D.J., Hewko C.D. (2024). Vegetative induction increases plant resistance to antagonistic insect frugivores. bioRxiv.

[5] Gols R. (2025). Tolerance to insect herbivory increases with progressing plant development. Plant Biol..

[6] Rusman Q., Lucas-Barbosa D., Hassan K., Poelman E.H. (2020). Plant ontogeny determines strength and associated plant fitness consequences of plant-mediated interactions between herbivores and flower visitors. J. Ecol..

[7] Lehndal L., Ågren J. (2015). Herbivory differentially affects plant fitness in three populations of the perennial herb *Lythrum salicaria* along a latitudinal gradient. PLoS ONE.

[8] McCall A.C., Irwin R.E. (2006). Florivory: The intersection of pollination and herbivory. Ecol. Lett..

[9] Boaventura M.G., Villamil N., Teixido A.L., Tito R., Vasconcelos H.L., Silveira F.A., Cornelissen T. (2022). Revisiting florivory: An integrative review and global patterns of a neglected interaction. New Phytol..

[10] Rodríguez-Morales D., Aguirre-Jaimes A., García-Franco J.G. (2024). Effects of florivory on floral visitors and reproductive success of Sagittaria lancifolia (Alismataceae) in a Mexican Wetland. Plants.

[11] Ghara M., Ewerhardy C., Yardeni G., Matzliach M., Sapir Y. (2017). Floral herbivory does not reduce pollination-mediated fitness in shelter rewarding Royal Irises. bioRxiv.

[12] Vega-Polanco M., Rodríguez-Islas L.A., Escalona-Domenech R.Y., Cruz-López L., Rojas J.C., Solís-Montero L. (2020). Does florivory affect the attraction of floral visitors to buzz-pollinated Solanum rostratum?. Arthropod-Plant Interact..

[13] Ye Z.M., Jin X.F., Wang Q.F., Yang C.F., Inouye D.W. (2017). Pollinators shift to nectar robbers when florivory occurs, with effects on reproductive success in *Iris bulleyana* (Iridaceae). Plant Biol..

[14] Martins J.K.S.S., Carneiro A., Souza L., Almeida-Cortez J. (2019). How pollinator visits are affected by flower damage and ants presence in *Ipomoea carnea* subs. *fistulosa* (Martius and Choise) (Convolvulaceae)?. Braz. J. Biol..

[15] Tsuji K., Ohgushi T. (2018). Florivory indirectly decreases the plant reproductive output through changes in pollinator attraction. Ecol. Evol..

[16] Cárdenas-Ramos D., Mandujano M.C. (2019). Florivory effects on pollinator preference and the reproductive output of a threatened living rock cactus, *Ariocarpus retusus* (Cactaceae). Haseltonia.

[17] Krupnick G.A., Weis A.E., Campbell D.R. (1999). The consequences of floral herbivory for pollinator service to *Isomeris arborea*. Ecology.

[18] 18, Mulligan G.A. (1972). Autogamy, allogamy, and pollination in some Canadian weeds. Can. J. Bot..

[19] Waddington K.D. (1976). Foraging patterns of halictid bees at flowers of *Convolvulus arvensis*. Psyche.

[20] Prokop P., Neupauerova D. (2014). Flower closure in the field bindweed (Convolvulus arvensis): A field test of the pollination hypothesis. Turk. J. Bot..

[21] Prokop P. (2024). Urban environment decreases pollinator availability, fertility, and prolongs anthesis in the field bindweed (*Convolvulus arvensis* Linnaeus, 1753). Plant Signal. Behav..

[22] Schaller N. (1993). The concept of agricultural sustainability. Agric. Ecosyst. Environ..

[23] Culhavi C.D., Manea D. (2011). Controlling *Convolvulus arvensis* L. in grain maize and winter wheat in Banat (Romania). Res. J. Agric. Sci..

[24] Carper A.L., Adler L.S., Irwin R.E. (2016). Effects of florivory on plant-pollinator interactions: Implications for male and female components of plant reproduction. Am. J. Bot..

[25] McCall A.C. (2008). Florivory affects pollinator visitation and female fitness *in Nemophila menziesii*. Oecologia.

[26] Mulligan G.A., Kevan P.G. (1973). Color, brightness, and other floral characteristics attracting insects to the blossoms of some Canadian weeds. Can. J. Bot..

[27] Prokop P., Zvaríková M., Ježová Z., Fedor P. (2020). Functional significance of flower orientation and green marks on tepals in the snowdrop *Galanthus nivalis* (Linnaeus, 1753). Plant Signal. Behav..

[28] 28, Jones P.L., Ryan M.J., Chittka L. (2015). The influence of past experience with flower reward quality on social learning in bumblebees. Anim. Behav..

[29] Møller A.P., Eriksson M. (1995). Pollinator preference for symmetrical flowers and sexual selection in plants. Oikos.

[30] Møller A.P., Sorci G. (1998). Insect preference for symmetrical artificial flowers. Oecologia.

[31] Goulson D., Cruise J.L., Sparrow K.R., Harris A.J., Park K.J., Tinsley M.C., Gilburn A.S. (2007). Choosing rewarding flowers; perceptual limitations and innate preferences influence decision making in bumblebees and honeybees. Behav. Ecol. Sociobiol..

[32] Mothershead K., Marquis R.J. (2000). Fitness impacts of herbivory through indirect effects on plant–pollinator interactions in *Oenothera macrocarpa*. Ecology.

[33] 33, Patiño S., Jeffree C., Grace J. (2002). The ecological role of orientation in tropical convolvulaceous flowers. Oecologia.

